# Chain-End Modifications and Sequence Arrangements of Antimicrobial Peptoids for Mediating Activity and Nano-Assembly

**DOI:** 10.3389/fchem.2020.00416

**Published:** 2020-05-21

**Authors:** Abshar Hasan, Varun Saxena, Valeria Castelletto, Georgina Zimbitas, Jani Seitsonen, Janne Ruokolainen, Lalit M. Pandey, Jan Sefcik, Ian W. Hamley, King Hang Aaron Lau

**Affiliations:** ^1^Department of Pure and Applied Chemistry, University of Strathclyde, Glasgow, United Kingdom; ^2^Department of Biosciences and Bioengineering, Indian Institute of Technology Guwahati, Guwahati, India; ^3^Department of Chemistry, University of Reading, Reading, United Kingdom; ^4^Department of Chemical and Process Engineering, University of Strathclyde, Glasgow, United Kingdom; ^5^Nanomicroscopy Center, Aalto University, Espoo, Finland

**Keywords:** self-assembly, micelles, antimicrobial peptide, peptoids, DLS, CAC, MIC

## Abstract

Poly(N-substituted glycine) “peptoids” are an interesting class of peptidomimics that can resist proteolysis and mimic naturally found antimicrobial peptides (AMPs), which exhibit wide spectrum activity against bacteria. This work investigates the possibility of modifying peptoid AMP mimics (AMPMs) with aliphatic lipid “tails” to generate “lipopeptoids” that can assemble into micellar nanostructures, and evaluates their antimicrobial activities. Two families of AMPMs with different distributions of hydrophobic and cationic residues were employed—one with a uniform repeating amphiphilicity, the other with a surfactant-like head-to-tail amphiphilicity. To further evaluate the interplay between self-assembly and activity, the lipopeptoids were variously modified at the AMPM chain ends with a diethylene glycol (EG_2_) and/or a cationic group (Nlys-Nlys dipeptoid) to adjust amphiphilicity and chain flexibility. Self-assembly was investigated by critical aggregation concentration (CAC) fluorescence assays and dynamic light scattering (DLS). The structure of a key species was also verified by small-angle X-ray scattering (SAXS) and cryo-electron microscopy (cryo-EM). To screen for antibacterial properties, we measured the minimum inhibitory concentrations (MIC) against *S. aureus, E. coli*, and *P. aeruginosa*. We found that certain combinations of lipid tail and AMPM sequences exhibit increased antibacterial activity (i.e., decreased MICs). Perhaps counter-intuitively, we were particularly interested in *increased* MICs in combination with low CACs. Concealing antimicrobial interactions due to packing of AMPMs in nano-assemblies could pave the way to AMPMs that may be “inert” even if unintentionally released and prevent microbes from gaining resistance to the lipopeptoids. Overall, incorporation of EG_2_ significantly improved lipopeptoids packing while the hydrophobic tail length was found to have a major influence over the MIC. One particular sequence, which we named C_15_-EG_2_-(kss)_4_, exhibited a very low CAC of 34 μM (0.0075 wt.%) and a significantly increased MIC above values for the unmodified AMPM. With the sequence design trends uncovered from this study, future work will focus on discovering more species such as C_15_-EG_2_-(kss)_4_ and on investigating release mechanisms and the potency of the released lipopeptoids.

**Graphical Abstract:**
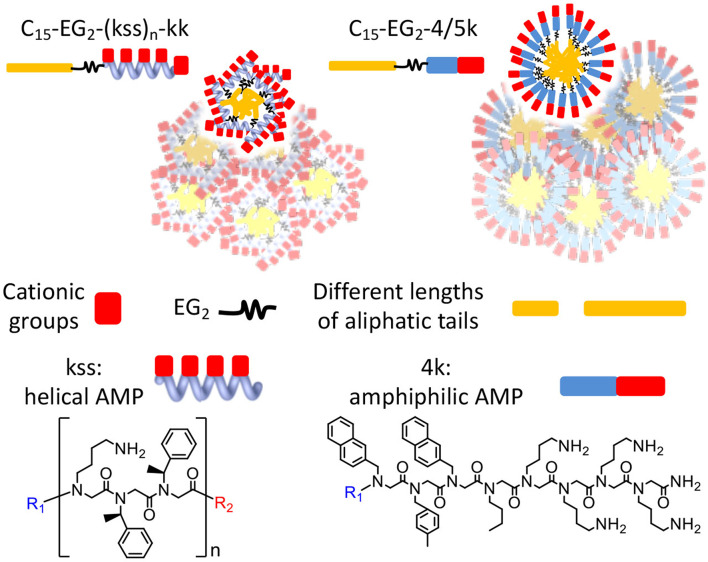
Chain end modifications of antimicrobial peptoids embodying different lengths and conformations result in lipopeptoids with various combinations of self-assembly and antimicrobial properties. A diethylene glycol linker and alternating cationic and hydrophobic residues are key to controlling self-assembly.

## Introduction

The past decades have witnessed a significant increase in antimicrobial resistance (AMR) causing a large burden on healthcare (Pendleton et al., 2013; Molchanova et al., 2017). Antibiotic drugs have enabled modern medicine by treating microbial infection. However, their specific mechanisms of binding to cellular molecular targets enables bacteria to develop resistance (Chen et al., 2014). Hence, new antimicrobials need to be developed, especially those that exhibit different or broad-spectrum bactericidal activity. For example, various peptides and polymer designs have been proposed to disrupt bacterial membranes by broadly utilizing electrostatic interactions with subsequent pore generation (Fukushima et al., 2012; Haney et al., 2017). Moreover, antimicrobial peptides (AMPs) expressed by many living species as a defense mechanism against other bacteria, fungi and viruses, have been proposed as an alternative to antibacterial and antifungal drugs (Zasloff, 2002; Raaijmakers et al., 2010; Zhao et al., 2017). However, peptides, especially oligomeric species exhibiting antimicrobial activity, are prone to biodegradation under the effect of bacterial and other extracellular proteases. To overcome such limitations, there is a significant drive to generate more stable peptidomimics (Fukushima et al., 2012; Jain et al., 2014; Molchanova et al., 2017).

Inspired by the diverse nanostructures and biofunctionality of natural peptides, peptide amphiphiles (or lipopeptides) with one or more lipid tails attached to an (amphiphilic) peptide head group have been a focus of biomaterials research. Depending on the peptide sequence and the length of the hydrophobic “tail,” these surfactant-like molecules can self-assemble into nanoassemblies such as micelles, nanofibrils, nanotapes, rods, vesicles, etc., (Hamley, 2015). Demonstrations of a range of applications in food safety and therapeutics, drug delivery, crop protection and other biomedical applications have been reported (De La Rica and Matsui, 2010; Zelzer and Ulijn, 2010; Hamley, 2015). However, as peptide materials, they do not address the problem of proteolytic degradation.

Peptoids are a recently developed class of peptidomimics (Simon et al., 1992). Their sidechains are attached to the amide nitrogen of N-substituted glycine residues, rather than to the backbone α-carbons as in peptides (Simon et al., 1992), thus preventing their recognition by proteases and enabling resistance against proteolysis. This sidechain shift also results in an achiral backbone and preclusion of inter- and intra-backbone hydrogen bonding, and hence backbone conformational flexibility is enhanced (Sun and Zuckermann, 2013; Lau, 2014). Bioactivity and formation of secondary and supramolecular structures are therefore tailored based predominantly on side-chain sequence selection (Czyzewski and Barron, 2008; Lau, 2014). A powerful advantage of peptoids is the “submonomer” solid phase synthesis approach for conveniently generating sequences with diverse natural and non-natural sidechains (Zuckermann et al., 1992; Lau, 2014; Lau et al., 2017). Antimicrobial and antifouling peptoid sequences (Statz et al., 2008; Lau et al., 2012; Lohan and Singh Bisht, 2013), anti-biofouling surfaces (Hasan et al., 2020), applications in drug discovery (Zuckermann and Kodadek, 2009), diagnosing amyloid proteins (Luo et al., 2013), etc., have all been reported. Peptoid self-assembly is a rapidly emerging area, and a number of groups have reported a variety of nanostructures, including micelles (Lau et al., 2017), nanotubes (Jin et al., 2018), and nanosheets (Robertson et al., 2016; Castelletto et al., 2019). However, the interplay between self-assembly and bioactivity is still less explored.

In this work, we report the design, synthesis and characterization of amphiphilic lipopeptoids as potential self-assembling antimicrobials. Previous work by Findlay et al. demonstrated the replacement of cationic lipopeptides with lipopeptoids without affecting their antimicrobial activities (Findlay et al., 2012). However, they did not emphasize the self-assembly of their lipopeptoids and their bioactivity thereafter. We have previously demonstrated the predictable formation of ultra-small and highly stable spherical micelles from “lipopeptoids” comprising of a lipid tail coupled to an amphiphilic peptoid oligomer (Lau et al., 2017). We have now generated a set of bioactive lipopeptoids by coupling existing high-potency peptoid AMPMs (antimicrobial peptide mimics), originally reported by the Barron (Chongsiriwatana et al., 2008) and Hansen (Ryge and Hansen, 2005) groups, to hydrophobic lipid tails of different lengths (i.e., C_8_, C_11_ and C_15_). Further insertions of ethylene glycol (EG_2_) at the N-terminus of the AMPM and a pair of cationic residues at the C-terminus were explored for controlling self-assembly and antibacterial behavior. Self-assembly was characterized by critical aggregation concentration (CAC) fluorescence assays and dynamic light scattering (DLS), supplemented by cryo-transmission electron microscope (cyro-TEM) and small angle X-ray scattering (SAXS). Minimum inhibitory concentration (MIC) assays against the Gram positive *Staphylococcus aureus* and Gram negative *Pseudomonas aeruginosa* and *Escherichia coli* were used to screen for changes in antimicrobial activity of our library compared to the native AMPMs. While it may be anticipated that specific sequences might be discovered to exhibit enhanced antimicrobial activity and/or propensity for self-assembly after chain end modifications, we were also interested in discovering sequences with *increased* MICs above their CACs. Such cases could indicate “packaging” of antimicrobial sequences into inactive nanostructured delivery vehicles, which could later be triggered for nanostructure disassembly and release of the lipopeptoids. This could provide an approach for preventing microbes from gaining exposure to the AMPMs if the lipopeptoids were unintentionally released into such an environment, thereby acquiring resistance.

## Materials and Methods

### Materials

All solvents and chemicals used (including HPLC-grade mobile phases) were purchased from Sigma-Aldrich UK, unless otherwise specified. Rink amide MBHA resin was bought from Merck, UK. Tert-butyl N-(4-aminobutyl) carbamate (NLys) and (1S)-1-phenylethylamine (Nspe) monomers were purchased from Apollo scientific, UK. Fmoc-amino-3,6 dioxaoctanoic acid was procured from FluroChem, UK.

### Lipopeptoids Synthesis

Peptoids were synthesized on resin manually or with an automated synthesizer (Prelude X, Gyros Protein Technologies) using a solid phase “submonomer” approach (Zuckermann et al., 1992; Lau, 2014; Lau et al., 2017). Briefly, the Fmoc protected rink amide resin was deprotected with 20% piperidine for 20 min, applied twice. Each residue was added by treatment to a mixture of bromoacetic acid (20 times excess) and diisopropyl-carbodiimide (18.5 times excess) for 15 min, followed by a halo-substitution reaction with appropriate primary amine submonomers. For the attachment of a di ethylene glycol linker, Fmoc-amino-3,6 dioxaoctanoic acid (1.8 mmol), and equivalent moles of HBTU ((2-(1H-benzotriazol-1-yl)-1,1,3,3-tetramethyluronium hexafluorophosphate) were reacted with the terminal amino group in the presence of N,N-diisopropylethylamine (DIPEA, 2.7 mmol). This reaction was carried out for 2 h at 37°C and repeated a second time for 4 h, to ensure coupling. Like resin Fmoc deprotection, EG_2_ N-terminal Fmoc deprotection was achieved using 20% piperidine for 20 min, applied twice. Different chain lengths of saturated fatty acids, i.e., pelargonic acid (C_8_), lauric acid (C_11_), and palmitic acid (C_15_) were subsequently coupled using the HBTU amide coupling as reported previously (Lau et al., 2017). After solid phase synthesis, the resin was cleaved and side chains were deprotected for 30 min using a standard TFA:TIPS:H_2_O cocktail (95:2.5:2.5 v/v/v). Excess TFA was removed using a rotary evaporator and the peptoid was precipitated from the oily product using diethyl ether. The collected material was dissolved in small amounts of 1:1 acetonitrile:water (ACN:H_2_O) to ease sample transfer, and further dried in a lyophilizer. Dried crude products were weighed and dissolved in ACN-H_2_O mixtures for preparative RP-HPLC (Dionex Ultimate 3000) with a C18 column (250 × 10 mm Phenomenex Jupiter). Fractions containing the pure product were identified by ESI-LC-MS (Agilent 1200 with a Poroshell C18 column coupled to an Agilent 6130 mass spectrometer) and by analytical RP-HPLC (Dionex P680) using a C18 column (250 × 4.6 mm “Nucleosil”) with a 30 min gradient of 5–95% acetonitrile (ACN) in water containing 0.1% TFA at 1 mL/min. The purified fractions of peptoids and lipopeptoids were later stored as aliquots at −20°C until further analysis. The analytical HPLC and ESI-MS data are shown in Figures S1–S5.

### Dynamic Light Scattering (DLS) Analysis

DLS measurements were carried out at room temperature (25°C) using an ALV/LSE-5004 instrument equipped with He-Ne laser (λ = 632.8 nm) at a detection angle of θ = 90° (Lau et al., 2017). Samples were prepared at a concentration of 0.4 wt.% in deionized water (DI) water and further filtered using 0.22 μm pore size surfactant-free cellulose acetate (SFCA) syringe filters (Sigma Aldrich, UK) to avoid particulate contamination.

### Cryogenic Transmission Electron Microscopy (Cryo-TEM)

Sizes of selected lipopeptoid nano-assemblies were verified using a field emission cryo-electron microscope (JEOL JEM3200FSC), operating at 200 kV and at −187°C, configured in bright field mode and zero loss energy filtering (omega type) with a slit width of 20 eV. Sizes of selected lipopeptoids nano-assemblies were measured from photographs recorded using a Gatan Ultrascan 4000 CCD camera. Vitrified specimens were prepared on Quantifoil 3.5/1 holey carbon copper grids (hole size of 3.5 μm). The grids were first plasma cleaned using a Gatan Solarus 9500 plasma cleaner and then transferred into the environmental chamber of an automated FEI Vitrobot device at room temperature and 100% humidity. Thereafter, 3 μL of the sample solution was applied on the grid, which was blotted twice for 5 s and then vitrified in a 1:1 mixture of liquid ethane and propane at a temperature of −180°C. The grids with vitrified sample solution were maintained at liquid nitrogen temperature and then cryo-transferred to the microscope.

### Small Angle X-Ray Scattering (SAXS)

SAXS experiments were performed on the bioSAXS beamline B21 at Diamond Light Source, U.K., using a previously established protocol (Castelletto et al., 2019). Briefly, solutions containing 1 wt.% peptoid were loaded into a set of PCR tubes fitted in the 96-well plate of the BioSAXS robot. From there, the robot loaded the solutions into a quartz capillary perpendicularly placed in the path of the incident X-ray beam. SAXS data, collected using a Dectris PILATUS 2 M detector at a fixed camera length of 3.9 m, is presented as scattering intensity vs. *q* = 4π sinθ /λ (2θ: scattering angle; wavelength: λ = 1 Å). See Supplementary Information for description of SAXS data fitting.

### Critical Aggregation Concentration (CAC) Assay

Nile red (NR) fluorescence was used to measure critical aggregation concentration (CAC) for the synthesized lipopeptoids. Lipopeptoids were dissolved at 2.5 × 10^−2^ to 1 wt.% in a dilute solution of Nile red (0.25 μg/100 mL, i.e. ~10 nM concentration) prepared in water. NR was excited at 552 nm and emission was recorded at room temperature in the range 550–750 nm using a 10.0 × 5.0 mm^2^ quartz cell (Jasco spectrofluorimeter FP-6500). The intensity of the peak centered around 610 nm is shown in the data presented in Section results and discussion.

### Antibacterial Analysis

Minimum inhibitory concentration (MIC) analysis was performed against Gram positive (*Staphylococcus aureus* (NCTC 4135), and Gram negative [*Escherichia coli* (ATCC 25922), and *Pseudomonas aeruginosa* (PA01)] strains. Analysis was performed in Mueller–Hinton (MH) broth using the broth microdilution method (CLSI M07-A10) in 96 well plates (CLSI, 2015). Bacterial strains (~5 × 10^5^ CFU/mL) with different concentrations of peptoids and lipopeptoids were incubated overnight at 37°C. Optical density (OD) was recorded at 600 nm using a plate reader (Infinite 200 Pro, Tecan). The MIC value (concentration for which the OD was halfway between the minimum and maximum values) was obtained by fitting the OD data to a sigmoidal model using Origin software, which also computed the standard deviation. Technical triplicates (*n* = 3) or more of samples at each concentration were analyzed.

## Results and Discussion

### Design of Lipopeptoids Based on (kss)_4_ and 4/5k Antimicrobial Sequences

The basic peptoid design follows our earlier work that attached a palmitic acid (C_15_) hydrophobic lipid tail to the N-terminus of oligopeptoids 6–8 residues long (Lau et al., 2017). These earlier oligopeptoids were typified by a short amphiphilic segment with alternating hydrophobic/hydrophilic residues and a cationic, highly soluble C-terminal Nlys-Nlys “kk” dipeptoid, inserted with the intention to increase water solubility. Self-assembly of the resulting lipopeptoids was strongly driven by the hydrophobic lipid tail and the relatively bulky, but water soluble oligopeptoid.

**Figure 1:**
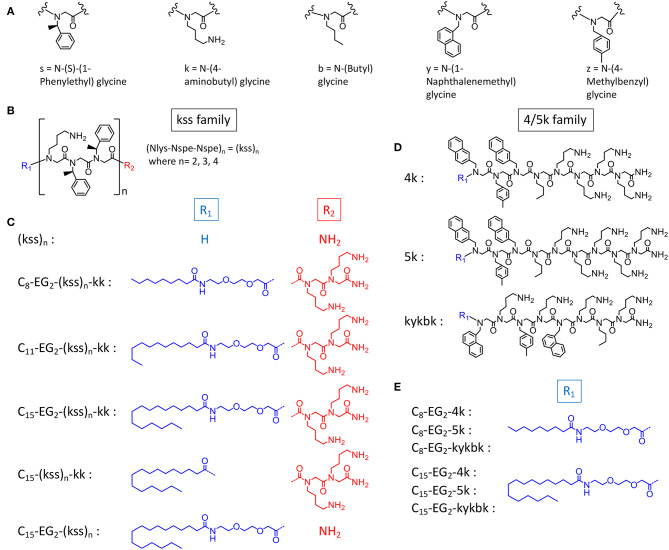
Chemical structures of **(A)** peptoid monomers, **(B)** the AMPM (kss)_n_, where *n* = 2, 3, or 4, **(C)** C- and N-terminal modifications of the kss family of lipopeptoids, **(D)** the Nlys-rich 4k, 5k, and kykbk AMPs from Hansen's work (Ryge and Hansen, 2005), and **(E)** the corresponding 4/5k family of lipopeptoids.

In this study, we postulate that nano-assemblies can likewise be formed from lipopeptoids prepared by attaching lipid tails to the chain ends (i.e., C- and N- termini) of known antimicrobial amphiphilic oligopeptoids (Figure 1). To investigate the scope of this approach, we included two families of antimicrobial peptoids (AMPMs) for modification, one reported by Barron et al. (Patch and Barron, 2003; Chongsiriwatana et al., 2008) and the other by Ryge and Hansen (2005). These AMPMs were chosen not only for their demonstrated effectiveness against a wide spectrum of bacteria but also for possessing different distributions of amphiphilicity along their sequences. Moreover, the two families exhibit different sequence distribution of cationic and hydrophobic residues. Barron group's helical AMPMs are typified by multiple repeats of an amphiphilic *N*lys-*N*spe-*N*spe trimeric motif, which we designated as “kss,” with the small letters k and s denoting Nlys and Nspe residues, respectively (Figures 1B,C). We selected three AMPMs from the Ryge and Hansen study consisting of several consecutive cationic Nlys residues at the C-terminus followed by a hydrophobic segment of similar length. The corresponding lipopeptoids are designated as the 4/5k family since one sequence has four consecutive C-terminal Nlys residues (4k), another has five (5k), and the third has an alternating amphiphilic C-terminal “kykbk” sequence (Figures 1D,E) (Ryge and Hansen, 2005).

To study how the AMPM sequences need to be balanced by the lipid tail and other components to obtain high assembly propensities, we included designs with different lengths of the hydrophobic aliphatic tail (C_8_, C_11_, C_15_). For the kss motif we also studied lipopeptoids with varying AMPM motif lengths [i.e., (kss)_n_, where *n* = 2, 3, 4]. While longer hydrophobic tails (C_8_, C_11_, C_15_) should promote assembly of hydrophobic cores, the effect of repeating the kss motif is less clear. However, it has been shown that longer kss sequences are more potent AMPMs (Czyzewski and Barron, 2008). In addition, analogous to our initial lipopeptoid report (Lau et al., 2017), we inserted the cationic C-terminal “kk” dipeptoid with the intention of creating a more water soluble “headgroup.” A new feature, a flexible diethylene glycol hydrophilic linker (EG_2_) is introduced between the AMPM and the lipid tail, since our earlier study indicated that flexibility along the sequence could promote assembly of more compact and stable micelles. The exact sequences, bioactivity and self-assembly parameters (i.e., MIC and CAC) of all designs are listed in Table 1.

**Table 1:** Characteristics of antimicrobial peptoids and lipopeptoid designs tested and measured values of CAC and MIC (see sections self-assembly of kss lipopeptoids to antibacterial MIC measurements for description).

**Compound name**	**Sequence**	**MW**	**CAC (μM)**	**Antimicrobial activity**		
				***S. aureus*^Ψ^(μM)**	E. coli^*^ (μM)	***P. aeruginosa*^#^ (μM)**
(kss)_2_	H-(Nlys-Nspe-Nspe)_2_-NH_2_	918	NA	41 ± 2	>100	44 ± 2
(kss)_3_	H-(Nlys-Nspe-Nspe)_3_-NH_2_	1,369	NA	2 ± 0.2	4 ± 0.2	15 ± 1
(kss)_4_	H-(Nlys-Nspe-Nspe)_4_-NH_2_	1,819	NA	4 ± 0.3	6 ± 0.4	16 ± 0.3
C_8_-EG_2_-(kss)_2_-kk	CH_3_(CH_2_)_7_-(OCH_2_CH_2_)_2_O)-(Nlys-Nspe-Nspe)_2_-Nlys-Nlys-NH_2_	1,459	200 ± 37	9 ± 1	18 ± 3	13 ± 3
C_8_-EG_2_-(kss)_3_-kk	CH_3_(CH_2_)_7_-(OCH_2_CH_2_)_2_O)-(Nlys-Nspe-Nspe)_3_-Nlys-Nlys-NH_2_	1,909	270 ± 30	3 ± 1	2 ± 0.3	6 ± 2
C_8_-EG_2_-(kss)_4_-kk	CH_3_(CH_2_)_7_-(OCH_2_CH_2_)_2_O)-(Nlys-Nspe-Nspe)_4_-Nlys-Nlys-NH_2_	2,360	290 ± 14	2 ± 0.1	2 ± 0.3	2 ± 0.4
C_11_-EG_2_-(kss)_2_-kk	CH_3_(CH_2_)_10_-(OCH_2_CH_2_)_2_O)-(Nlys-Nspe-Nspe)_2_-Nlys-Nlys-NH_2_	1,501	200 ± 17	7 ± 1	9 ± 1	19 ± 1
C_11_-EG_2_-(kss)_3_-kk	CH_3_(CH_2_)_10_-(OCH_2_CH_2_)_2_O)-(Nlys-Nspe-Nspe)_3_-Nlys-Nlys-NH_2_	1,951	310 ± 38	1 ± 0.1	3 ± 0.2	15 ± 0.4
C_11_-EG_2_-(kss)_4_-kk	CH_3_(CH_2_)_10_-(OCH_2_CH_2_)_2_O)-(Nlys-Nspe-Nspe)_4_-Nlys-Nlys-NH_2_	2,402	270 ± 31	2 ± 0.2	7 ± 0.1	14 ± 1
C_15_-(kss)_4_-kk	CH_3_(CH_2_)_14_-(Nlys-Nspe-Nspe)_4_-NlysNlys-NH_2_	2,315	300 ± 25	>100	>100	>100
C_15_-EG_2_-(kss)_4_-kk	CH_3_(CH_2_)_14_-(OCH_2_CH_2_)_2_O)-(Nlys-Nspe-Nspe)_4_-Nlys-Nlys-NH_2_	2,458	180 ± 11	36 ± 5	44 ± 5	>100
C_15_-EG_2_-(kss)_4_	CH_3_(CH_2_)_14_-(OCH_2_CH_2_)_2_O)-(Nlys-Nspe-Nspe)_4_-NH_2_	2,201	38 ± 3	50 ± 1	>100	>100
4k	H-(Nnme-Nmbe-Nnme-Nbut-(Nlys)_4_)-NH_2_	1,198	NA	7 ± 0.1	7 ± 0.3	19 ± 0.4
5k	H-(Nnme-Nmbe-Nnme-Nbut-(Nlys)_5_)-NH_2_	1,326	NA	8 ± 0.1	7 ± 0.2	33 ± 1
Kykbk	H-(Nnme-Nlys-Nmbe-Nlys-Nnme-Nlys-Nbut-Nlys)-NH_2_	1,198	NA	9 ± 0.4	15 ± 1	20 ± 1
C_8_-EG_2_-4k	CH_3_(CH_2_)_7_-(OCH_2_CH_2_)_2_O)-(Nnme-Nmbe-Nnme-Nbut-(Nlys)_4_)-NH_2_	1,483	2500 ± 350	18 ± 3	17 ± 1	16 ± 2
C_8_-EG_2_-5k	CH_3_(CH_2_)_7_-(OCH_2_CH_2_)_2_O)-(Nnme-Nmbe-Nnme-Nbut-(Nlys)_5_)-NH_2_	1,611	1600 ± 390	8 ± 0.3	74 ± 4	41 ± 2
C_8_-EG_2_-kykbk	CH_3_(CH_2_)_7_-(OCH_2_CH_2_)_2_O)-(Nnme-Nlys-Nmbe-Nlys-Nnme-Nlys-Nbut-Nlys)-NH_2_	1,483	1900 ± 200	2 ± 0.2	11 ± 4	15 ± 3
C_15_-EG_2_-4k	CH_3_(CH_2_)_14_-(OCH_2_CH_2_)_2_O)-(Nnme-Nmbe-Nnme-Nbut-(Nlys)_4_)-NH_2_	1,581	320 ± 24	>100	>100	>100
C_15_-EG_2_-5k	CH_3_(CH_2_)_14_-(OCH_2_CH_2_)_2_O)-(Nnme-Nmbe-Nnme-Nbut-(Nlys)_5_)-NH_2_	1,709	360 ± 25	>100	75 ± 10	>100
C_15_-EG_2_-kykbk	CH_3_(CH_2_)_14_-(OCH_2_CH_2_)_2_O)-(Nnme-Nlys-Nmbe-Nlys-Nnme-Nlys-Nbut-Nlys)-NH_2_	1,581	410 ± 32	72 ± 20	35 ± 18	>100

*NA, Not Applicable*;

Ψ *NCTC 4135*;

* *ATCC 25922*;

# *PA01*.

### Self-Assembly of kss Lipopeptoids

We first examined with a CAC assay whether coupling a hydrophobic tail to AMPMs and inserting the kk and EG_2_ components is a viable design for generating self-assembling lipopeptoids. The CAC measures the concentration above which hydrophobic pockets (e.g., lipophilic cores of micelles) are formed, and lower CACs indicate higher propensities of self-assembly. In our experiments, the CAC is indicated by an inflection in the increase in emission intensity of a lipophilic fluorophore tracer (Nile Red) that emits more strongly when partitioned into hydrophobic lower dielectric environments (see Figure S6 for an example analysis).

**Figure 2:**
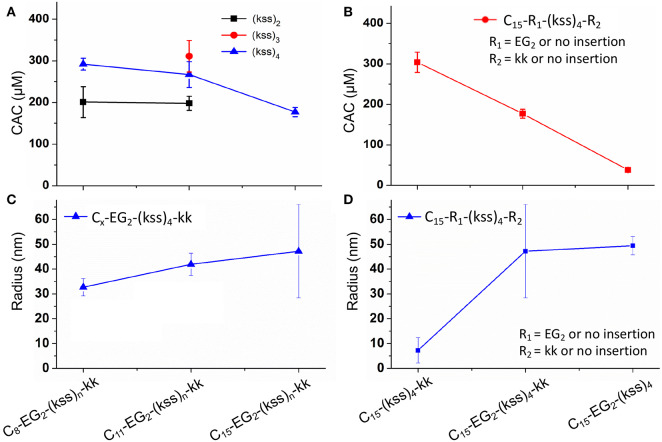
Plots of CACs **(A,B)** and hydrodynamic radii **(C,D)** of the kss lipopeptoids studied. The left column **(A,C)** shows the data for increasing tail length from C_8_, C_11_ to C_15_, for different lengths of the (kss)_n_ motif, and the right column **(B,D)** shows the influence of the kk cationic headgroup and the EG_2_ linker, based on the (kss)_4_ motif and a C_15_ tail.

Taking the highly potent (kss)_4_ sequence as a template, we generated a C_x_-EG_2_-(kss)_4_kk series of lipopeptoids, with *x* = 8, 11, 15, to examine how the length of the lipidic tail would influence the propensity of assembly. As shown in Figure 2A, we observed CACs of 200~220 μM (a.k.a. 0.50~0.52 mg/mL, or 0.050~0.052 wt.%), with increasing lengths of the hydrophobic tail corresponding to lower CACs. These CACs are similar to the lowest values obtained in our first reports of lipopeptoid assembly (0.03~0.1 wt.%) (Lau et al., 2017) and are, in fact, relatively low for lipopeptides in general. DLS measurements of nanostructures with hydrodynamic radius (R_H_) in the range of ~40 nm (Figures 2C,D) further verified that our basic lipopeptoids design is viable.

**Figure 3:**
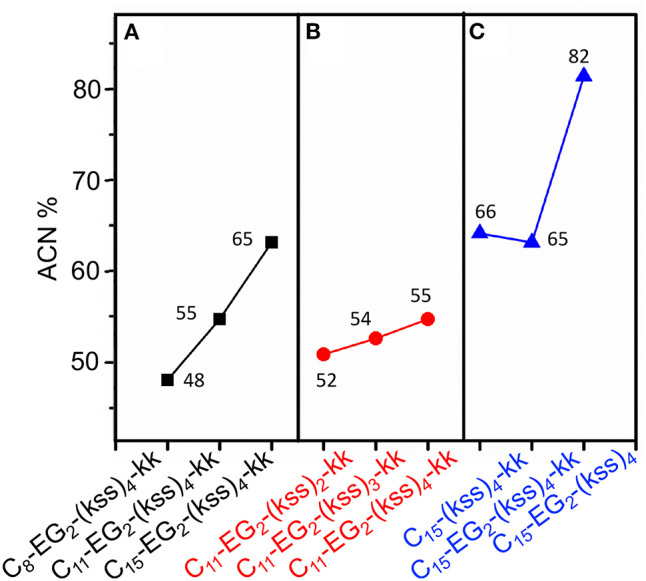
Analytical RP-HPLC results of elution off a C_18_ column over a 5-95% ACN gradient for the kss family of lipopeptoids. **(A)** The length of the lipid tail *C*_x_ is varied: *x* = 8, 11, 15. **(B)** The length of the antimicrobial sequence (kss)_n_ is changed: *n* = 2, 3, 4. **(C)** The arrangement of the EG_2_ and kk segments is varied. The values labeling each data point is the elution ACN% of individual sequences.

Interestingly, effectively doubling the hydrophobic tail length (C_8_ vs. C_15_) only decreased CAC slightly by 10%. The corresponding increase in molecular weight of the lipopeptoids was from 2,361 to 2,459 g/mol, a 4% difference in mass, whereas the corresponding *R*_H_ increases from 30 to 45 nm for C_8_ to C_15_ was statistically marginal. However, there was a significant increase in RP-HPLC retention on a hydrophobic column (increase from 48 to 65% ACN from C_8_ to C_15_) (Figure 3A). The above indicate that the lipid tail was not the main determinant of self-assembly in this system, with self-assembly being likely determined by a combination of other components, i.e., EG_2_, (kss)_n_ motif, and the kk headgroup.

We next examined the assembly process with different lengths of the central antimicrobial (kss)_n_ motif (*n* = 2, 3), using an intermediate C_11_ tail as an example (Figure 2A). In this case, the CAC became either moderately lower (198 μM/0.29 mg/mL for the shortest C_11_-EG_2_-(kss)_2_-kk) or higher (267 μM//0.52 mg/mL for C_11_-EG_2_-(kss)_3_-kk) than C_11_-EG_2_-(kss)_4_-kk. Although there was no apparent trend in CAC, the HPLC retention of the lipopeptoids (in %ACN) did increase with increasing number of kss motif repeats (Figure 3B). Therefore, longer (kss)_n_ segments could contribute to an increased ability of the lipopeptoid to partition in a non-polar phase, as expected, but self-assembly was mostly driven by other effects. Since the tail length was also seen to weakly contribute, we next examined the arrangement of the lipopeptoids' design components.

We returned to the C_15_-EG_2_-(kss)_4_-kk base sequence to examine the influence of the kk and EG_2_ insertions. The (kss)_4_ length was chosen because it has been reported to give the highest antimicrobial activity (Chongsiriwatana et al., 2008), and we chose the longest C_15_ tail because it is seen to confer the highest propensity to self-assemble, complementing our intention to minimize the CAC. Figure 2B shows that removing the EG_2_ linker to obtain C_15_-(kss)_4_-kk resulted in assembly only at a much higher CAC of 304 μM (0.71 mg/mL). On the other hand, removing the charged kk head group to form C_15_-EG_2_-(kss)_4_ dramatically decreased CAC to a very low 38 μM (0.075 mg/mL, or 0.0075 wt.%; Figure S6).

The decrease in CAC with removal of kk (i.e., C_15_-EG_2_-(kss)_4_) was accompanied by a greatly increased HPLC elution to 82% ACN from 65% ACN (C_15_-EG_2_-(kss)_4_-kk; Figure 3C) while removing EG_2_ but retaining kk caused essentially no change in the retention (66% ACN). Ordinarily, peptoids/peptides eluting at these relatively high ACN contents would precipitate in aqueous buffers. This was not observed, which is consistent with solubility of the material enabled by micellization.

Formation of nano-assemblies from C_15_-EG_2_-(kss)_4_ was further confirmed by DLS observations, which here gave R_H_ of ca. 50 nm (Figure 2D). To corroborate DLS results complementary structural characterizations by SAXS and cryo-TEM were also performed. SAXS indicates the presence of nano-assemblies 24 ± 5 nm in radius (Figure S7A and Table S1). TEM shows that some of these assemblies actually comprise of non-compact aggregates as small as ca. 5 nm diameter (Figure S7B). Irregularity in shape would have contributed to relatively larger hydrodynamic sizes measured by DLS. On the other hand, the smallest features seen by TEM could have been missed by DLS and SAXS since scattering signals scale significantly with particle size.

In our initial report of lipopeptoid self-assembly (Lau et al., 2017), we speculated that bending along the lipopeptoid backbone could enable insertion of lipid tails toward a hydrophobic central core while allowing the amphiphilic peptoid sequence to lie parallel to the micelles' outer surface, with ionizable groups facing the external aqueous environment. The increase in CAC with removal of EG_2_ is consistent with this view, since assembly would be less favorable without the EG_2_ linker enhancing flexibility between the aliphatic tail and the peptoid sequence. The much lower CAC after removing the charged kk “headgroup” is also in agreement with the aforementioned hypothesis, since removing this headgroup would reduce charge repulsion between peptoids and enhance uniformity of the micelle's outer shell.

### Self-Assembly of 4/5k Lipopeptoids

**Figure 4:**
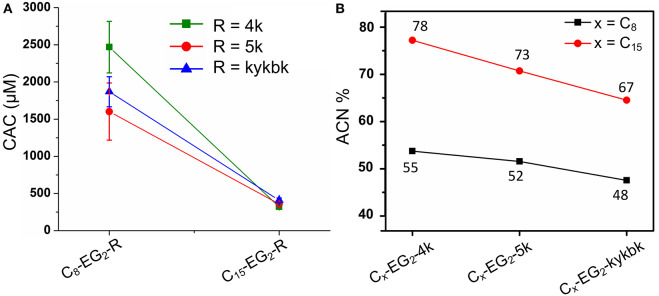
CAC **(A)** and analytical RP-HPLC **(B)** results of the 4/5k family of lipopeptoids with tail lengths of C_8_ and C_15_. HPLC elution was carried out using a C_18_ column and a 5–95% ACN gradient.

Lipopeptoids of the 4/5k family generally exhibited a lower assembly propensity than the kss lipopeptoids, and the aliphatic tail length had a much greater influence on their self-assembly. Figure 4A shows that 4/5k lipopeptoids with C_15_ tails all exhibited a CAC around 300~400 μM (0.50~0.65 mg/mL), while those with C_8_ tails exhibited CAC at least 3-times higher (i.e. low assembly propensity), from 1,600 to 2,500 μM (2.4~3.7 mg/mL). Accordingly, analytical HPLC showed that the much lower CACs, associated with the longer C_15_ tails, were correlated with substantially higher retention and hydrophobicity (from 67 to 78 %ACN; Figure 4B) than that exhibited by the C_8_ series (from 48 to 55 %ACN). However, differences between the 4/5k peptoid sequences appear to play a less important role in the CAC (i.e., assembly process) at each individual tail length. Although CAC at the C_15_ tail length did indeed decrease from 410 ± 32 to 320 ± 24 μM according to increasing HPLC retention from 67 to 78 ACN% (counting from kykbk, 5k, to 4k), the trend at the C_8_ tail length is less clear—the most hydrophobic 4k actually resulted in the highest CAC (2,500 ± 350 μM) and the kykbk and 5k sequences generated statistically similar CAC despite their significantly different retentions (i.e., 1,900 ± 200/1,600 ± 390 μM and 48/52 %ACN for kykbk/5k).

In comparison, the tail length and the kss repeat length for the kss family of lipopeptoids had a limited influence on the CAC (Figures 2A,B).

**Figure 5:**
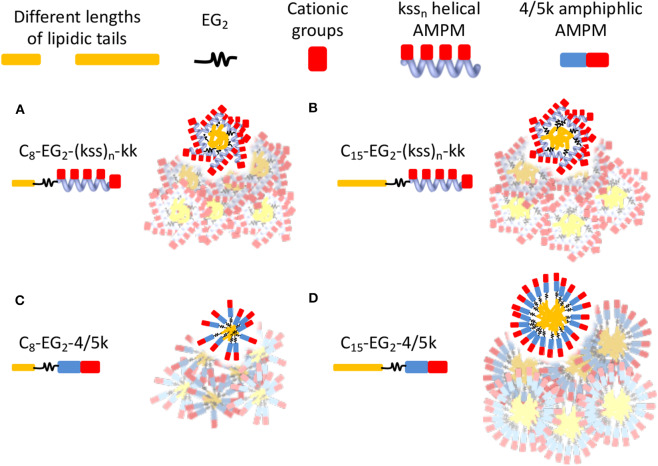
Schematics illustrating *hypothetical* differences and similarities in lipopeptoid arrangements in nano-assemblies due to different conformations and hydrophobicity distributions. Water-soluble cationic groups on one face of helical kss_n_ may constrain changes in hydrophobic interactions and size of nano-assemblies, even if different lengths of lipidic tails are used **(A,B)**. The head-tail surfactant-like 4/5k may lead to more conventional assemblies controlled by length of lipidic tails **(C,D)**. The low assembly propensity (high CAC) of C_8_-EG_2_-4/5k is indicated by their irregular, looser packing. The overlapping micelles illustrate the possibility of aggregates in generating some of the large sizes measured by DLS. Different micelle morphologies may also be possible but are not shown.

A significant difference between the two families is the distribution of hydrophobic and cationic residues along the antimicrobial peptoid sequences. The kss sequences express a regular repeating kss trimer motif that induces a helical conformation placing all the cationic sidechains on one face of this helix. In contrast, the 4/5k sequences have a pronounced head-to-tail switch in hydrophobicity, with many water soluble cationic Nlys residues placed toward the C-terminus (thus no additional kk headgroup is added to the 4/5k lipopeptoids) and multiple hydrophobic residues placed toward the N-terminus (Figure 1D). As such, the 4/5k lipopeptoids resemble conventional surfactants, with a defined soluble head group and a hydrophobic tail. Indeed, it seems plausible that the peptoid conformations within the self-assemblies would be different between the two families, with the conventional surfactant configuration of the 4/5k lipopeptoids allowing for more control of the CAC via the tail length changes. These differences between the overall distribution in hydrophobic sequence regions and our hypothesis of the subsequent effects on assembly are summarized in Figure 5. Overall, a very large contribution of the hydrophobic tail is needed to sway the assembly behavior, whereas differences in conformations and hydrophobicity distributions of the two series (i.e., helical kss, head-tail amphiphilic 4/5k series and kk head group, flexible EG_2_ linker) appear to strongly contribute to the assembly process.

### Antibacterial MIC Measurements

**Figure 6:**
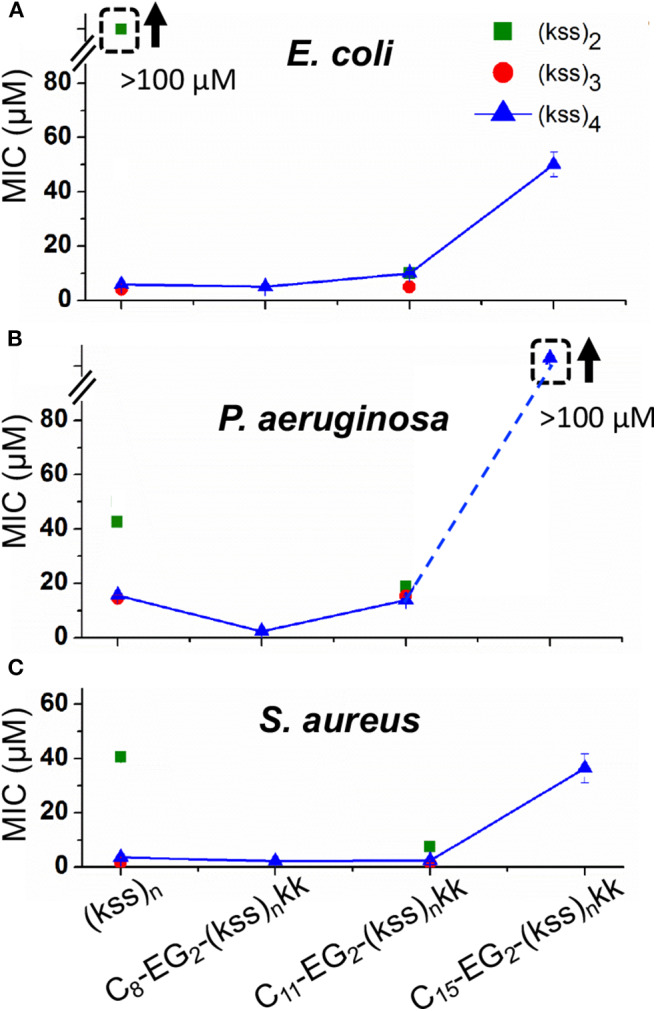
MICs against *E. coli* **(A)**, *P. aeruginosa*. **(B)**, and *S. aureus* **(C)** measured for C_8_-EG_2_-(kss)_n_-kk lipopeptoids with increasing tail length C_8_, C_11_, and C_15_, and different lengths of the (kss)_n_ motif. Values measured for “unfunctionalized” (kss)_n_ peptoids are also shown. The legend in panel A applies to all data. The error bars denote ± 1 SD (standard deviation). Bounded boxes with an up arrow denote that no significant inhibition was observed up to the maximum tested 100 μM—the actual MICs could be significantly higher. Dashed lines highlight the corresponding data points in a series.

We measured the minimum inhibitory concentration (MICs) of our lipopeptoids for the Gram positive *Staphylococcus aureus* and the Gram negative *Escherichia coli* and *Pseudomonas aeruginosa*. The results for C_x_-EG_2_-(kss)_n_-kk series are shown in Figure 6. The bacteria here are presumed to be interacting with individually dissolved lipopeptoids as the MICs measured are all below the CACs obtained (Figure 2).

Three main effects were observed. First, the MICs for the (kss)_3_ and (kss)_4_ lipopeptoids at the C_8_ and C_11_ tail lengths were largely unchanged compared to the native unmodified AMPM, providing evidence that these AMPMs are amenable to chain end modifications. This opens the door to adapting these sequences for different applications, such as the present case of micellar self-assembly. Second, cases were observed where the lipopeptoids can become more active (decreased MIC). Specifically, C_11_-EG_2_-(kss)_2_-kk exhibited lower MICs than their respective native sequences against all strains tested (i.e., 40~50 vs. 10~20 μM). The C_8_-EG_2_-(kss)_4_-kk also exhibited a slightly lower MIC against *P. aeruginosa* (16 vs. 5 μM). Third, the MICs at the C_15_ tail length increased significantly for all strains tested.

The increase in antimicrobial activity (decrease in MIC) after N-terminal modification for (kss)_2_ is interesting, as this short length is the least effective version of the kss AMPM reported. Addition of a lipophilic tail generates a more surfactant-like molecule and presumably assisted in membrane interactions, an effect that had been reported (Kapoor et al., 2011). However, for an AMPM that already has a length suitable for membrane interactions (i.e., the 12-mer (kss)_4_), addition of a lipid tail (e.g., C_15_-EG_2_-(kss)_4_-kk) did, in fact, interfere with its activity.

**Figure 7:**
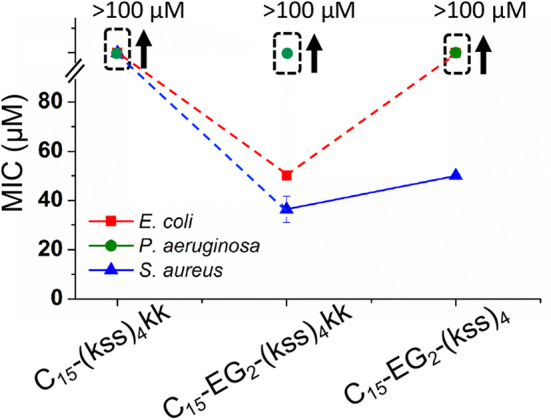
MICs against *E. coli, P. aeruginosa*, and *S. aureus* for (kss)_4_ lipopeptoids with C_15_ tails but without either the kk or EG_2_ components. The error bars denote ± 1 *SD*. Bounded boxes with an up arrow denote that no significant inhibition was observed up to the maximum tested 100 μM—the actual MICs could be significantly higher. Dashed lines highlight the corresponding data points in a series.

Figure 7 shows the effect on the MIC after removing EG_2_ or kk units while maintaining the C_15_ tail length. It is seen that C_15_-(kss)_4_-kk exhibited a dramatically increased MIC (lowered activity) above 100 μM for all bacteria tested, higher than for the range observed for C_15_-EG_2_-(kss)_4_-kk (Figure 6), and much higher than the 5~16 μM MICs of the native (kss)_4_. As such, the EG_2_ linker appears to be necessary to separate the lipid tail from the (kss)_4_ sequence for bacterial interactions. However, removing kk For C_15_- EG_2_-(kss)_4_ also caused increases in MIC, to >100 μM against both *E. coli* and *P. aeruginosa*, and ~50 μM against *S. aureus*. For this sequence, the CAC at 34 μM (Figure 2B) was lower than the MIC. Thus, these increased MICs could be an *apparent* effect due to packing of the AMPMs in nano-assemblies. Future studies will be required to investigate release mechanisms and the corresponding activities for those assemblies at concentrations above the CAC and below the apparent MIC.

**Figure 8:**
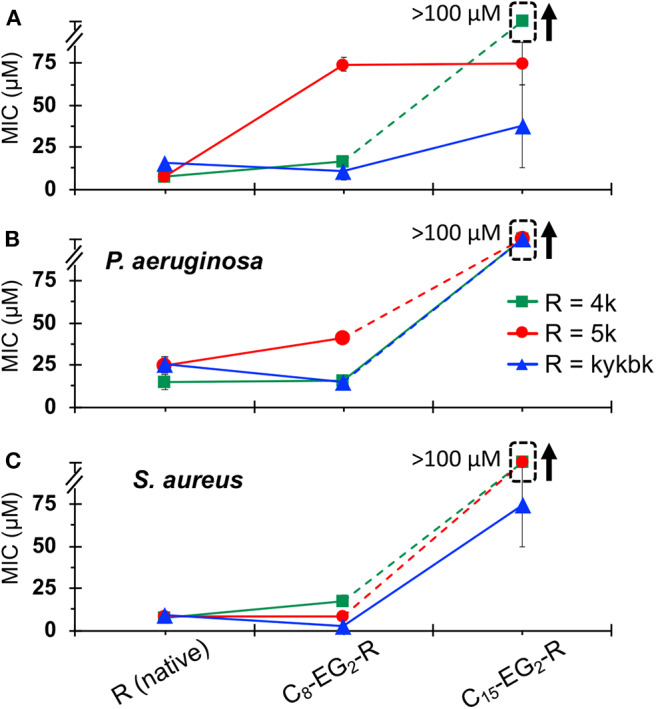
MICs against *E. coli* **(A)**, *P. aeruginosa*. **(B)**, and *S. aureus* **(C)** for 4/5k lipopeptoids with C_8_ and C_15_ tails compared with the native AMPM sequence. The error bars denote ± 1 *SD*. Bounded boxes with an up arrow denote that no significant inhibition was observed up to the maximum tested 100 μM—the actual MICs could be significantly higher. Dashed lines highlight the corresponding data points in a series.

The MICs for the 4/5k family of lipopeptoids are shown in Figure 8. Given the results for the kss lipopeptoids, the EG_2_ linker was always retained. Moreover, given the very high CACs of the 4/5k lipopeptoids, it is assumed that the interactions observed can all be assigned to individual chains, especially for the C_8_ series. Overall, no significant effect was observed after modifying the N-terminus with the shorter C_8_ tail, except for the 5k sequence against the Gram-negative *E*. *coli* and *P. aeruginosa*, for which the MIC increased. Secondly, lengthening the tail to C_15_ significantly increased the MIC in general, in many cases to above 100 μM. This paralleled the MIC increases for C_15_-EG_2_-(kss)_4_-kk (Figure 6). At the same time, given the ability of the C_15_ tail to decrease the CAC for 4/5k lipopeptoids, compared to the C_8_ tails (Figure 4), it would appear that the assembly-promoting properties of the C_15_-EG_2_ combination (Figure 2B) also interfered, in general, with antimicrobial activity.

## Discussion and Conclusion

This work investigates the interplay between self-assembly and antimicrobial activity for a series of lipopeptoids based on modifying the C- and N-terminal ends of existing AMPM antimicrobial peptoids. The effects of coupling a diethylene glycol “EG_2_” linker and lipid tails of varying lengths to the N-terminus, and a “kk” di-Nlys cationic headgroup to the C-terminus, were evaluated.

Lipopeptoids based on the “kss” family of helical AMPMs exhibited low CACs, generally around 200~400 μM (0.05~0.1 wt.%). These values are low for relatively small self-assembling species such as our lipopeptoids and indicate a strong propensity for self-assembly. Surprisingly, the length of the hydrophobic tail was found to only have a slight effect on assembly, and the solubilizing kk headgroup actually increased the CAC. However, an EG_2_ greatly enhanced self-assembly, and a very low CAC of 38 μM (0.0075 wt.%) was found for a C_15_-EG_2_-(kss)_4_ design. For 4/5k lipopeptoids, the kk headgroup is an intrinsic part of the antimicrobial sequence and the tail length became a controlling influence on assembly. Although we carried out our CAC characterization in water, fundamental thermodynamic forces generally drive micellar systems to lower CACs (i.e., higher assembly propensity) in higher buffer/salt conditions. However, instabilities resulting from uncontrolled aggregation may result in practice (Moreira and Firoozabadi, 2010; Palladino and Ragone, 2011).

Overall, the high assembly propensity of C_15_-EG_2_-(kss)_4_ could be associated with the unconventional linking (via use of a flexible EG_2_ unit) of a hydrophobic tail to a helical kss peptoid presenting cationic sidechains segregated to one side of the helix. We speculate that this design could enable an arrangement of kss peptoids parallel to the surfaces of micelles overshadowing the contribution of different lipid tail lengths to self-assembly. In contrast, markedly different assembly properties were observed with the head-to-tail amphiphilicity of 4/5k peptoids, which follow the expected lipid tail length dependencies.

The antimicrobial activities of the lipopeptoids, on the other hand, depended significantly on the hydrophobic tail length for both the kss and 4/5k designs. Many of our lipopeptoids with a C_8_ or C_11_ tail either preserved, or even decreased, the MICs compared to the native sequences. However, MICs increased significantly with a C_15_ tail. In evaluating activity, lowered MICs compared to the unmodified AMPM would be of interest if the lipopeptoid were interacting as individual molecules, as would be the case at concentrations below the CAC. Given the high potency of the AMPMs studied (10^0^ ~ 10^1^ μM), almost all the lipopeptoids we generated exhibited CACs (≥10^2^ μM) above their MICs (10^0^ ~ 10^2^ μM). Thus, most of our lipopeptoids would be interacting as solubilized species in the unassembled state.

We were also interested in *increases* in the MIC in combination with a low CAC. We hypothesized that packing of AMPMs into molecularly large nano-assemblies would interfere with regular membrane interactions necessary for activity, and hence induce an *apparent* increase in MIC. This would pave the way to AMPMs (and analogous AMP designs) that may remain “inert” in the environment, even if unintentionally released, and hence prevent microbes from gaining active exposure and developing resistance. The sequence C_15_-EG_2_-(kss)_4_ satisfied these criteria, exhibiting a CAC of 38 μM (0.0075 wt.%) combined with an MIC of 40 μM against the Gram-positive *S. aureus* and above 100 μM against the Gram-negative *E. coli* and *P. aeruginosa* strains tested. Further physical characterization by SAXS, TEM and DLS showed corresponding nano-assemblies ~40 nm in radius. Based on the trends observed in the current study, future work will be aimed at discovering more species with properties similar to or better than C_15_-EG_2_-(kss)_4_, and at investigating release mechanisms and the potency of the released sequences.

## Data Availability Statement

All datasets generated for this study are included in the article/Supplementary Material.

## Author Contributions

AH synthesized peptoids and all the peptoid designs and performed HPLC, LC-MS, MIC, and CAC experiments and analysis. VS synthesized selected peptoids and performed MIC experiments and analysis. VC and IH performed and analyzed SAXS results. GZ and JSef performed DLS measurements and analysis. JSei and JR cryo-EM and analyzed data. LP and KL co-supervised AH and VS. KL and AH conceptualized the work and wrote the manuscript.

## Conflict of Interest

The authors declare that the research was conducted in the absence of any commercial or financial relationships that could be construed as a potential conflict of interest.
