# Supplementary material for: Chain-End Modifications and Sequence Arrangements of Antimicrobial Peptoids for Mediating Activity and Nano-Assembly
Source: Front Chem. 2020 May 21;8:416. doi: 10.3389/fchem.2020.00416 (PMC7253723; doi:10.3389/fchem.2020.00416)
Supplement: Supplementary file 1 [file Data_Sheet_1.PDF]

Supplementary information

Chain-End Modifications and Arrangements of an Antimicrobial Peptoid Motif for Mediating Potency, Toxicity, and Nano-Assembly

Abshar Hasan[1,2,†], Varun Saxena[1,2], Valeria Castelletto[3], Georgina Zimbitas[4], Jani Seitsonen[5], Janne Ruokolainen[5], Lalit M. Pandey[2], Jan Sefcik[4], Ian W. Hamley[3], King Hang Aaron Lau[1]*

[1]Department of Pure & Applied Chemistry, University of Strathclyde, 295 Cathedral Street, Glasgow G1 1XL, UK

[2]Department of Biosciences and Bioengineering, Indian Institute of Technology Guwahati, Assam, 781039, India

[3]Department of Chemistry, University of Reading, Reading, RG6 6AD, UK

[4]Department of Chemical and Process Engineering, University of Strathclyde, 75 Montrose Street, Glasgow, G1 1XJ, UK

[5]Nanomicroscopy Center, Aalto University, Puumiehenkuja 2, FIN-02150 Espoo, Finland

Corresponding author: King Hang Aaron Lau; Email: aaron.lau@strath.ac.uk

1. Supplementary figures:

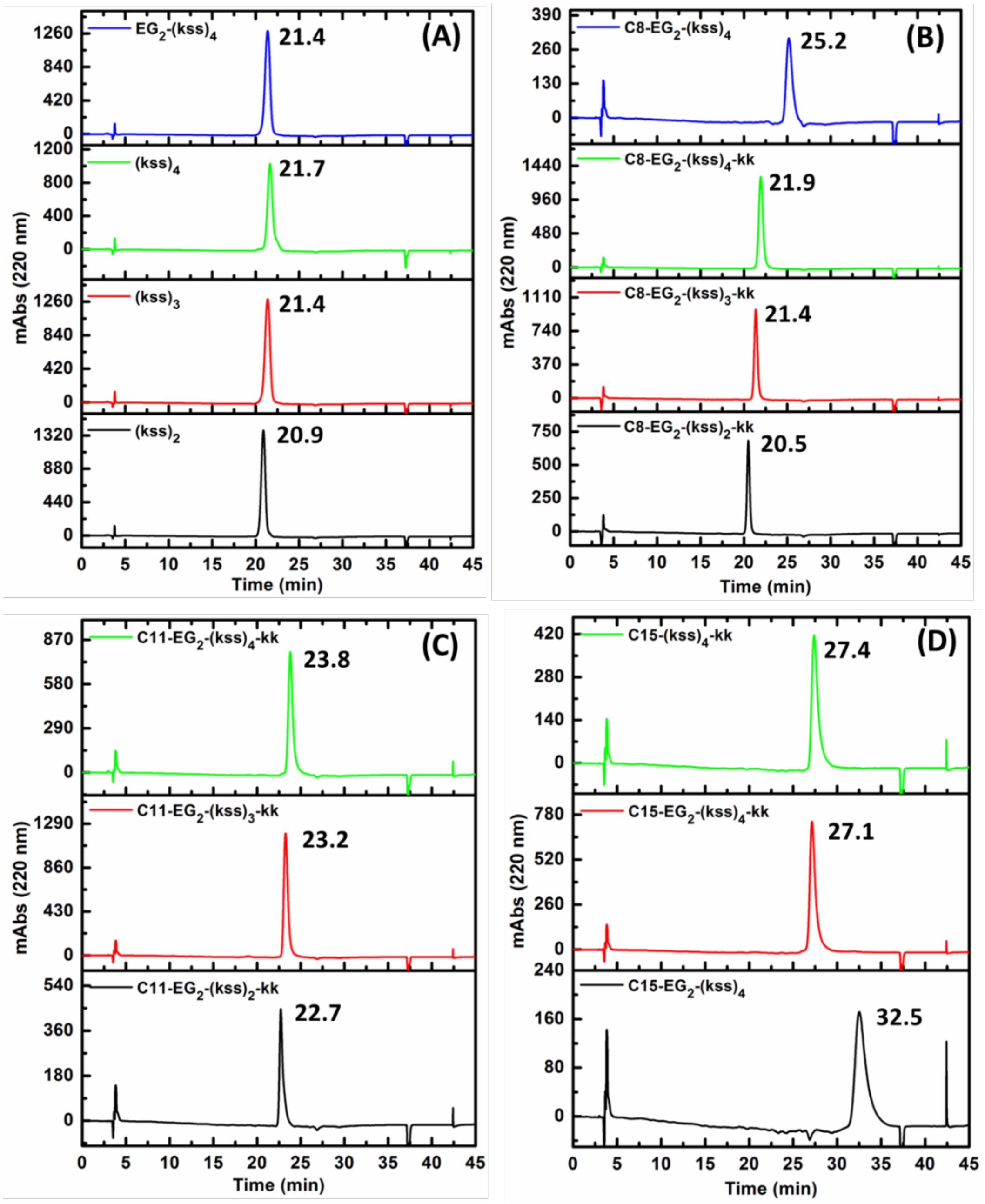

Figure S1. Analytical RP-HPLC chromatograms using a C18 column and UV-absorbance measured at 220 nm for purified fractions of (A) $(kss)_n$ peptoids, (B) C_8, (C) C_{11} modified lipopeptoids, and (D) C_{15} alkylated $(kss)_4$ based lipopeptoids. (See Table 1 and Figure 1 in the main text for explanation of the abbreviated peptoid names.) The 5-95 ACN% gradient begins at ca. 7 min and ends 30 min later at ca. 37 min (indicated by a disturbance). We expect some variability in the injection time, autosampler and injection volume which could have resulted in a slight uncertainty of the elution time. The graphs are included with the purpose of showing the purity and trend of synthesized molecules rather than for identification.

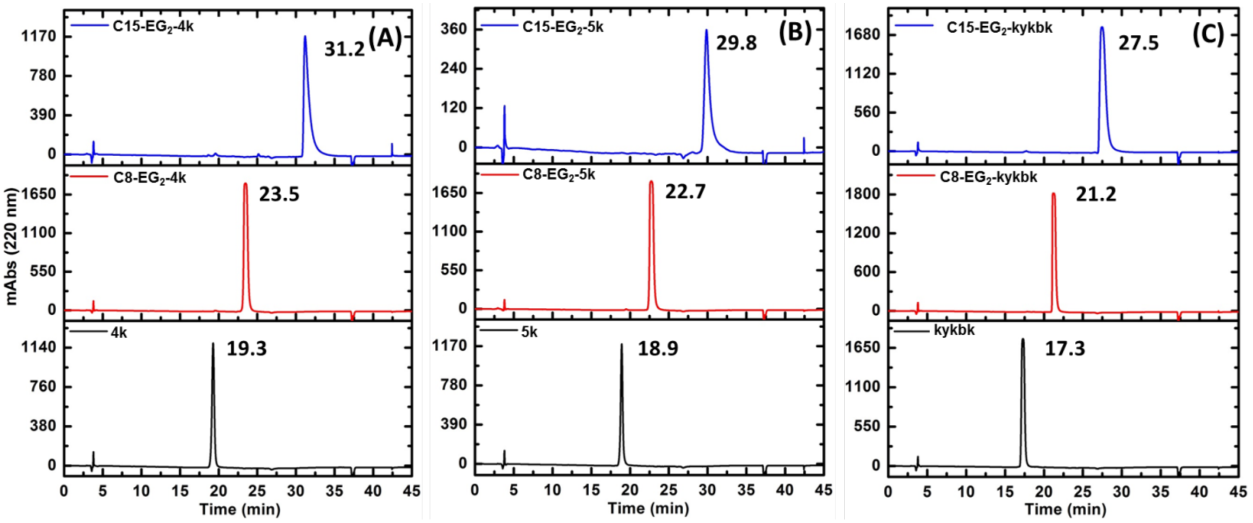

Figure S2. Analytical RP-HPLC chromatograms using a C18 column and UV-absorbance measured at 220 nm for purified fractions of (A) 4k, (B) 5k, and (C) kykbk peptoids along with their respective C_8 and C_{11} tailed lipopeptoids (see Table 1 and Figure 1 in the main text for explanation of the abbreviated peptoid names). The 5-95 ACN% gradient begins at ca. 7 min and ends 30 min later at ca. 37 min (indicated by a disturbance).

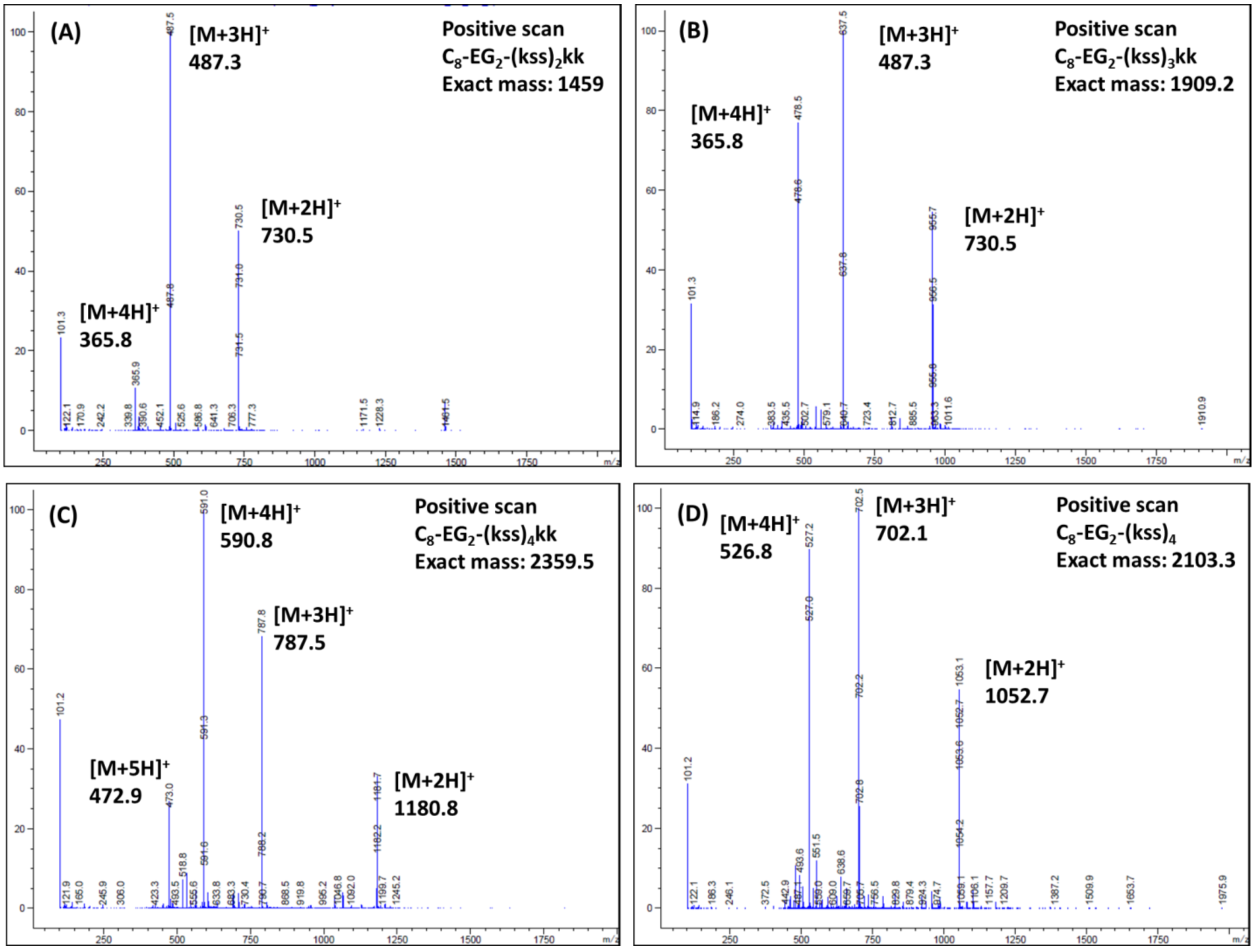

Figure S3. LCMS spectra (counts vs m/z) exhibiting positive ion mode for (A) C_8-EG_2-$(kss)_2kk$, (B) C_8-EG_2-$(kss)_3kk$, (C) C_8-EG_2-$(kss)_4kk$, and (D) C_8-EG_2-$(kss)_4$. See Table 1 and Figure 1 in the main text for explanation of the abbreviated peptoid names.

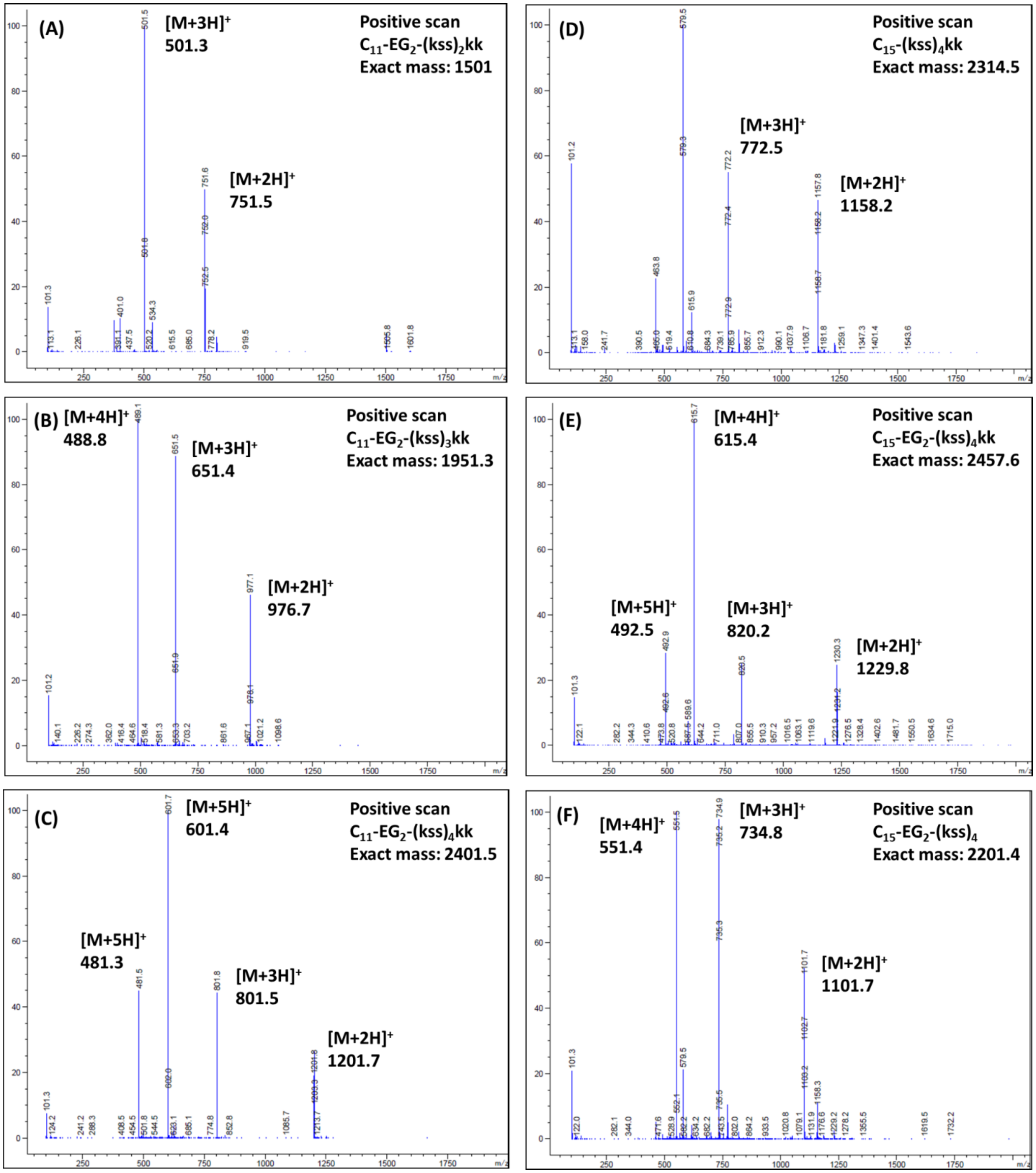

Figure S4. LCMS spectra (counts vs m/z) exhibiting positive ion mode for (A) C_{11}-EG_2-$(kss)_2$kk, (B) C_{11}-EG_2-$(kss)_3$kk, (C) C_{11}-EG_2-$(kss)_4$kk, (D) C_{15}-$(kss)_4$kk, (E) C_{15}-EG_2-$(kss)_4$kk, and (F) C_{15}-EG_2-$(kss)_4$. See Table 1 and Figure 1 in the main text for explanation of the abbreviated peptoid names.

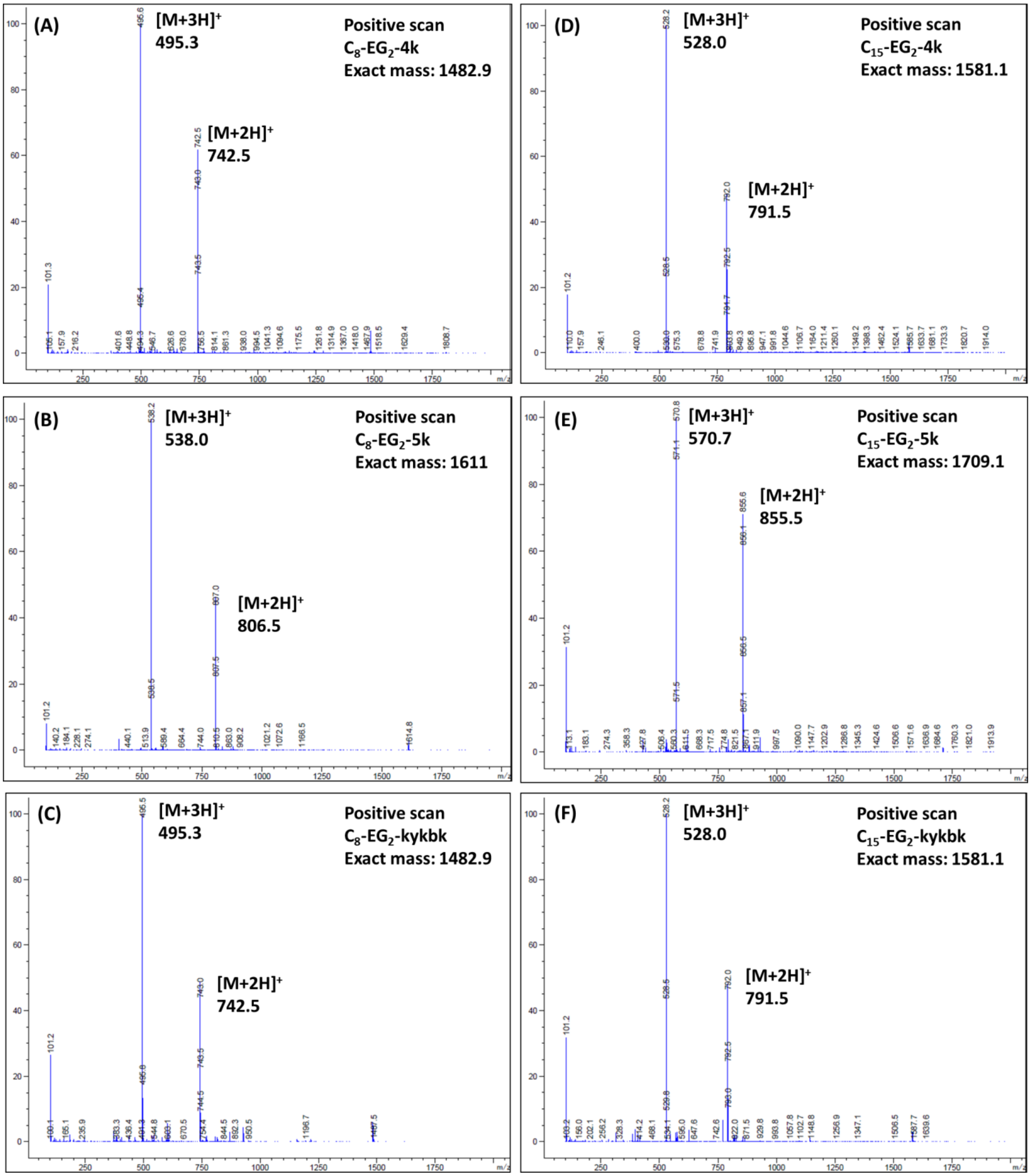

Figure S5. LCMS spectra (counts vs m/z) exhibiting positive ion mode for (A) C^8-EG_2-4k, (B) C_8-EG_2-5k, (C) C_8-EG_2-kykbk, (D) C_{15}-EG_2-4k, (E) C_{15}-EG_2-5k, and (F) C_{15}-EG_2-kykbk. See Table 1 and Figure 1 in the main text for explanation of the abbreviated peptoid names.

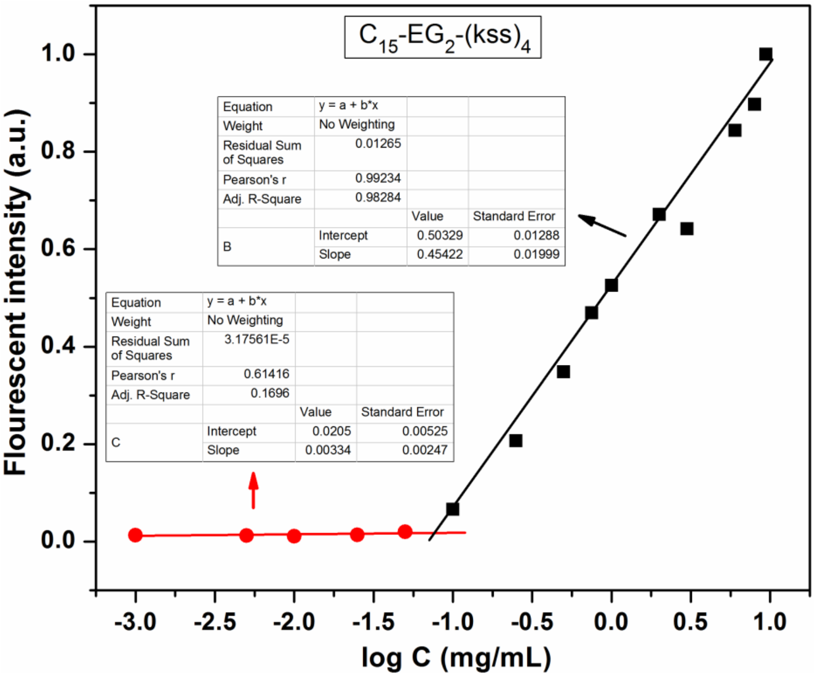

Figure S6. Nile Red fluorescence intensity plotted against log concentration of C_{15}-EG_2-$(kss)_4$ as an example to show how the CAC was calculated. The intensities were normalized to the highest values recorded at 10 mg/mL lipopeptoid. Intensities vs. log concentration were linearly fitted in Origin software separately for data points below and above the CAC, the distinction of which is easily observable via visual inspection. The intercept of the trends is then calculated from the fitted slopes and intercept parameters of the fits to obtain the CAC. The min-max errors of the regression were used to calculate the uncertainty.

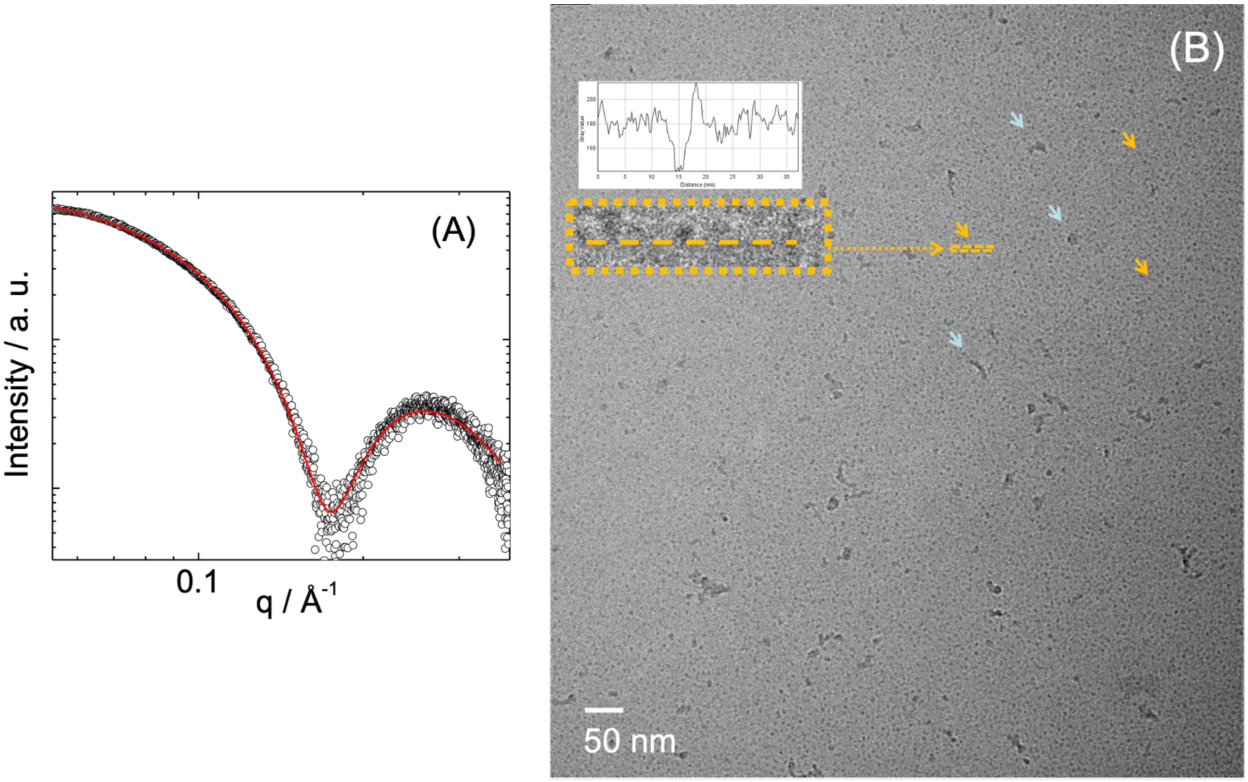

Figure S7. (A) SAXS form factor data of C_{15}-EG_2-(kss)$_4$ dissolved at 2 wt% in water (symbols) and fitted using a spherical core-shell model (red traces) by SASfit software. The fitting parameters are shown below in Table S1. (B) Cryo-TEM images for 2 wt% C_{15}-EG_2-(kss)$_4$ in water. Light blue and orange arrows point to some example individual micelles ~5 nm wide and micellar clusters up to ~50 nm wide respectively. The inset in (B) shows one of the single micellar objects in an expanded view. The accompanying intensity profile shows an easily recognizable ~5 nm wide minimum corresponding to a micelle along the line shown.

Table S1. Parameters obtained from the fitting of the SAXS data for 2 wt% C_{15}-EG_2-(kss)$_4$ in Figure 7A, using a spherical shell model.[1] We assume that the inner core of the micelle hosts more of denser C_{15} chains while the (kss)$_4$ block is mainly to the corona of the micelle.

**Parameter**	**C_{15}-EG_2-(kss)$_4$**
Inner radius, R and polydispersity ΔR (Å)	18.2 ± 4.6
Shell thickness, t (Å)	6.2
Core scattering contrast, η_c	1.06 x 10^{-5}
Relative shell scattering contrast, η_s	-4.5
Background	-1.75 x 10^{-4}

References

(1) Breßler, I.; Kohlbrecher, J.; Thünemann, A. F. SASfit: a tool for small-angle scattering data analysis using a library of analytical expressions. *J. Appl. Cryst.* **2015,** *48*, 1587-1598.
